# Potential Medical Applications of Chitooligosaccharides

**DOI:** 10.3390/polym14173558

**Published:** 2022-08-29

**Authors:** Sukumaran Anil

**Affiliations:** 1 Oral Health Institute, Department of Dentistry, Hamad Medical Corporation, Qatar University, Doha 3050, Qatar; asukumaran1@hamad.qa; Tel.: +974-50406670; 2 Pushpagiri Research Centre, Pushpagiri Institute of Medical Sciences and Research Centre (PIMS&RC), Thiruvalla, Pathanamthitta 689101, Kerala, India

**Keywords:** chitooligosaccharides, chitin, chitosan, anti-inflammatory, antioxidant, tissue engineering, wound healing, antimicrobial, drug delivery, antitumor

## Abstract

Chitooligosaccharides, also known as chitosan oligomers or chitooligomers, are made up of chitosan with a degree of polymerization (DP) that is less than 20 and an average molecular weight (MW) that is lower than 3.9 kDa. COS can be produced through enzymatic conversions using chitinases, physical and chemical applications, or a combination of these strategies. COS is of significant interest for pharmacological and medical applications due to its increased water solubility and non-toxicity, with a wide range of bioactivities, including antibacterial, anti-inflammatory, anti-obesity, neuroprotective, anticancer, and antioxidant effects. This review aims to outline the recent advances and potential applications of COS in various diseases and conditions based on the available literature, mainly from preclinical research. The prospects of further in vivo studies and translational research on COS in the medical field are highlighted.

## 1. Introduction

Chitin, a mucopolysaccharide, is produced by many living organisms and is usually present in a complex with other polysaccharides and proteins in insects, crustaceans, arachnids, myriapods, nematodes, algae, and fungi [1]. Chitin is a linear polysaccharide composed of (1 → 4) linked 2-acetamido-2-deoxy-*β*-d-glucopyranosyl units and occurs naturally in three polymorphic forms with different orientations of the microfibrils, known as *α*-, *β*-, and *γ*-chitin [2]. Chitin has been the focus of numerous therapeutic uses in addition to serving as a precursor for producing chitosan and chitooligosaccharides. Chitin has several uses in food, agriculture, wastewater treatment, textiles, microbiology, nanotechnology, chemistry, and material science [3]. It is biodegradable and is also considered as a promising biomaterial for tissue engineering and stem cell technologies [4].

Chitosan [poly-(*β*-1/4)-2-amino-2-deoxy-D-glucopyranose] is a natural nontoxic linear polysaccharide biopolymer produced through the deacetylation of chitin [5]. Commercial chitosan is made by deacetylating naturally occurring chitin and is used in dietary supplements, organic fertilizers, and cosmetics [6,7]. Chitin and chitosan can be distinguished based on the degree of acetylation of the D-glucosamine units. Chitin includes over 70% acetylated units, whereas chitosan contains less than 30% acetylation. In the presence of organic acids, including formic acid, acetic acid, and ascorbic acid, chitosan forms salt and becomes water soluble [8]. Chitosan possesses three reactive functional groups, including an amino- or N-acetamide group and two primary and secondary hydroxyl groups at the C-2, C-3, and C-6 positions. The amino- or N-acetamide groups distinguish the structure and physicochemical properties of various chitosans [9]. The fraction of N-acetylated residues (FA), degree of polymerization (DP), molecular weight (MW), MW distribution, and pattern or sequence of N-acetylation (PA) can be used to classify chitosan [10].

Chitosans with a DP of less than 20 and an average MW of less than 3.9 kDa are called chitooligosaccharides (COSs), chitosan oligomers, or chitooligomers [11,12]. Their characteristics, such as low molecular weight, low polymerization degree, and high water solubility, are superior to those of chitin and chitosan [11]. COS has a variety of biological activities and many potential uses in multiple fields, such as medicine, cosmetics, food, and agriculture [13]. In addition, chemical methods using acid, hydrogen peroxide (H_2_O_2_)_,_ or sodium nitrite (NaNO_2_) are also used to extract COS. There are multiple ways to obtain chitosan oligomers. These methods are categorized as enzymatic, physical, or chemical depolymerizations [14]. Chemical methods using acid [15,16], H2O2 [17], or NaNO2 [18] physical methods, such as hydrothermal [19], microwave [20], ultra-sonication [21], and gamma rays [22]. Among the chemical methods for the hydrolysis of chitosan, acid hydrolysis is probably the best known. The enzymatic depolymerization of chitosan is characterized by the enzymes’ selective cleavage of chitosan glycosidic bonds [23].

While hetero-chitooligosaccharides combine oligomers with varying DP, deacetylation degree (DD), and acetylation patterns (position of the N-acetyl residues in the chain), homo-chitooligosaccharides are oligomers made exclusively of either glucosamine (GlcN) or N-Acetylglucosamine (GlcNAc) units [3,24]. The attractive bioactive properties of COS make them suitable for various biological applications. A low molecular weight (1.5 kDa) results in aqueous solubility across a broad pH range and easy absorption through epithelial cells [10]. The physical and biological activities of chitosan and its oligomers are governed primarily by the DP, MW, and DD [3,25,26]. The most significant property of COS is its water solubility or solubility in physiological pH due to the freely accessible amino groups on the shorter chains [27]. All heterogeneous COS with a DP < 10 and DD between 50 and 100 % are completely soluble over the pH range extending from neutral to slightly alkaline, whereas commercially available chitosan with a higher DP precipitates out at such pH values [28].

COS exhibits superior solubility over a more extended pH range and at relatively higher concentrations than the corresponding chitosan, exhibiting exciting biological properties. Depending on their size, they are also soluble in other solvents, such as dimethyl sulfoxide, dimethylformamide, water, and alcohol. There has been a growing interest in modifying these oligomers to expand their applications. A low molecular weight (1.5 kDa) confers water solubility across a broad pH range and the capacity to be swiftly absorbed by epithelial cells. A wide variety of cells, including neuronal, embryonic, and bone-marrow-derived stem cells, can grow in COS with a degree of deacetylation (DD) > 85%, which is more compatible with cell growth than chitosan with lower DD levels [29]. The fabrication of compatible membrane materials, in which hydrogels are the most thoroughly investigated, is a key biomedical use of COS. Hydrogels are frequently used for therapeutic purposes, including tissue engineering and drug delivery, due to their propensity to expand in aqueous and biological fluids [30].

This review aims to provide a broad overview of chitooligosaccharides, their methods of preparation, and a comprehensive account of the preclinical and laboratory studies conducted to date on its application in the medical field. Based on the available preliminary information, future attention is being paid to translational studies to apply this preliminary information in clinical practice. The importance of focusing on translational studies in the future in order to apply the preliminary information in medical practice is highlighted.

## 2. Structure of Chitooligosaccharides

**Figure 1:**
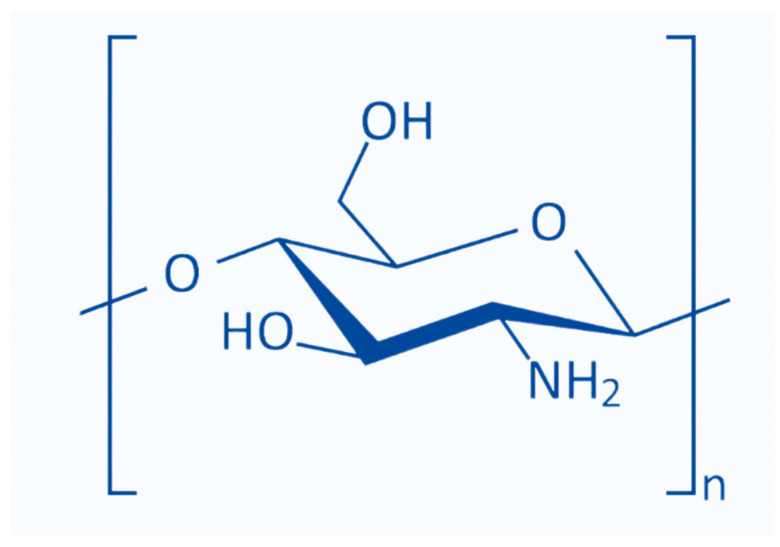
Basic chemical structure of N-deacetylated chitooligosaccharides. (Redrawn from the references: Liaqat and Eltem 2018 [3]; Naveed et al., 2019 [34]).

Chitosans with degrees of polymerization (DP) < 20 and an average molecular weight of 3900 Da are chitosan oligomers, chitooligomers, and chitooligosaccharides [12]. COS is synthesized by depolymerizing chitin or chitosan through acid hydrolysis, physical hydrolysis, and enzymatic degradation [11] (Figure 1). Chitooligosaccharides are composed of co-oligomers of *N*-acetyl-D glucosamine and D-glucosamine, usually prepared through the partial hydrolysis and deacetylation of chitin and chitosan [31]. The DD of COS, which is related to the molar units of GlcN on its backbone, depends on the DD of chitosan used as the starting material, determining the molecular weight distribution and N-acetylation pattern [32]. COS shares three common functional reactive groups that are directly responsible for inducing and enhancing its various biological effects. These reactive functional groups include the amino/acetamido group, and the primary and secondary hydroxyl groups, at the C-2, C-3, and C-6 positions, respectively, as well as the glycosidic bond at the β-(1→4) link, which is the link between the *N*-glucosamine units [33].

## 3. Preparation of Chitooligosaccharides

**Figure 2:**
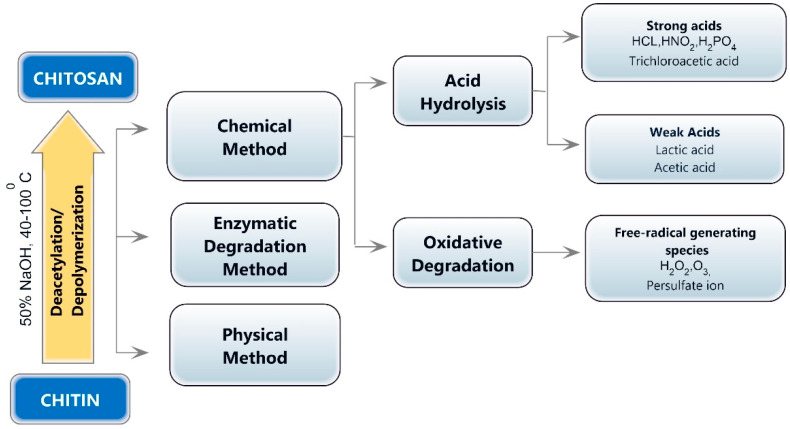
Various methods of chitooligosaccharide production.

The depolymerization of chitin or chitosan and the production of chitooligosaccharides can be accomplished using physical (microwave treatment, ultraviolet radiation, and ultrasonic treatment), chemical (hydrogen peroxide oxidation and acid hydrolysis), enzymatic, electrochemical, and composite degradation methods [10]. Ultrasonication, hydrothermal treatment, hydrodynamic cavitation, electromagnetic, gamma rays, and microwave irradiation are the physical methods for chitin or chitosan hydrolysis [35,36,37]. Chemical methods for producing chitooligosaccharides include degradative methods, such as breaking down chitin or chitosan with a strong acid or oxidation, and synthesis methods [38]. Chemical approaches for synthesizing chitooligosaccharides produce low yields and necessitate protection/deprotection processes, the employment of hazardous chemicals, the production of hazardous waste, high costs, and the generation of a large quantity of monomers [39]. The chemical degradation process is easy to employ, but the relative molecular weight of the degradation products is widely dispersed, making separation and purification challenging. In addition, the amount of reagents used is considerable, and the post-treatment is complicated. Acid and oxidative degradation are the two main processes involved in the chemical decomposition of COS (Figure 2).

### 3.1. Acid Hydrolysis of Chitosan 

Chitosan can be hydrolyzed using hydrochloric acid, acid with electrolytes, nitrous acid, phosphoric acid, and hydrofluoric acid, as well as oxidative and reductive techniques involving hydrogen peroxide or persulfate [11,40]. The MW and DD of COS products are highly influenced by the acid concentration, temperature, and treatment time [11]. The most commonly used acid for obtaining COS is hydrochloric acid (HCl). At higher temperatures, concentrated hydrochloric acid degrades chitosan directly to form a chitooligomer and increases the product’s DD. Jia and Shen [41] used 85% phosphoric acid to obtain water-soluble chitosan under optimal conditions at 60 degrees Celsius. Degradation by strong acids is difficult to control, the process is complex, and the process yields a lower quantity of CO. Weak acids, such as formic acid, acetic acid, and nitrous acid, can also be used to degrade chitosan. Nitrous acid also breaks down GlcN residues into 2,5-anhydromannose. A small amount of the amino group damage results from the GlcN ring being rearranged and released in this reaction, but GlcN is eventually repaired by sodium borohydride (NaBH4) [42].

### 3.2. Enzymatic Hydrolysis

**Figure 3:**
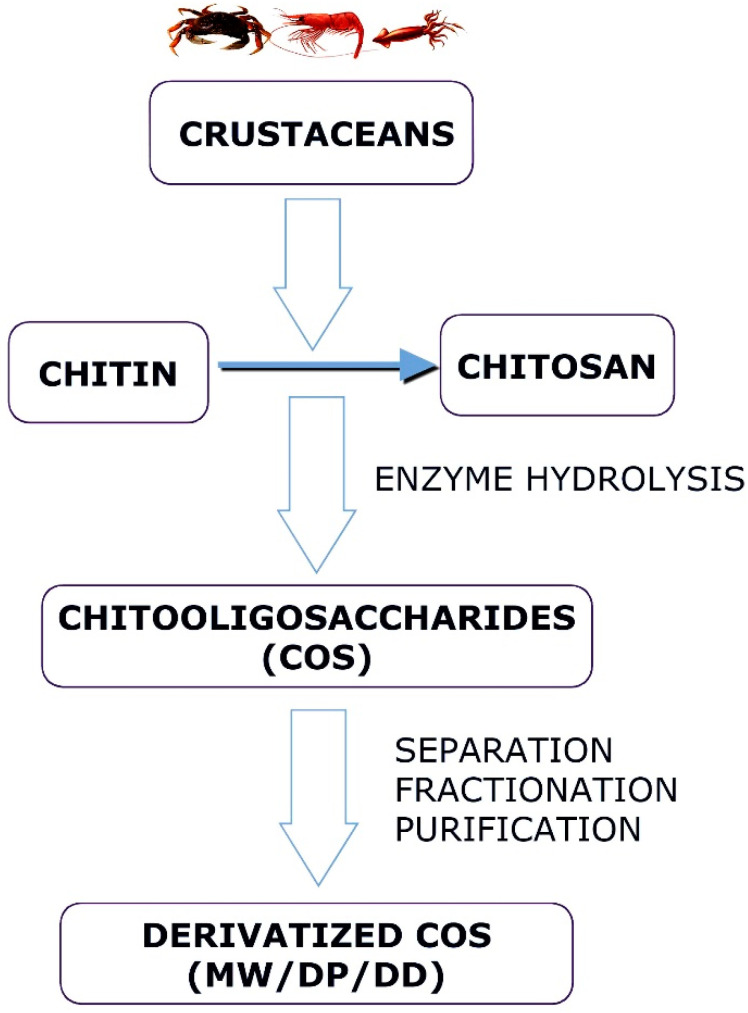
Schematic diagram showing the major steps in obtaining Chitooligosaccharides (enzymatic synthesis).

Enzymatic chitosan hydrolysis is superior to chemical reagent-catalyzed chitosan hydrolysis regarding its predictability and controllability [11,43]. The enzymatic procedures are precise and involve minimal chemical alteration of the products (Figure 3). Additionally, enzyme-catalyzed reactions are controllable, producing COS with specific chemical properties by modifying production procedures, such as enzyme concentrations, pH, temperature, and reaction time [44]. Several glycoside hydrolases composed of specialized enzymes, such as chitinases and chitosanases, can catalyze the enzymatic hydrolysis of chitin and chitosan [45]. Enzymatic hydrolysis can also be performed using nonspecific enzymes, including cellulase, amylase, lipase, and pectinase, with higher yields and lower costs [46,47]. Many factors can affect the outcome of an enzymatic depolymerization reaction. In manufacturing chitooligosaccharides from chitin or chitosan, the choice of enzyme and substrate exert a significant effect [38]. COS exhibits diverse biological activities, which depend on their molecular weight and DD compared to chitosan with a high molecular weight. The most efficient method for obtaining longer chains of COS is to use enzymes, and this method has been effectively used to manufacture COS spanning from dimers (DP 2) to octamers (DP 8) [48]. Cellulose cellulase demonstrated the best hydrolytic ability among the six nonspecific enzymes employed to degrade chitosan [49]. The enzymatic hydrolysis method necessitates optimal conditions for a reaction, and the raw materials’ structural properties may also impact the hydrolysis rate [50].

Several nonspecific enzymes, including proteolytic enzymes, such as papain, pepsin, and pronase, were used to produce COS from chitosan. Depolymerization was achieved using immobilized papain and chitosan. The advantage of immobilized papain was that it could be retrieved after the process, and the retrieval enzyme could be reused, despite the low yield and molecular weight of low-molar-mass chitosans (LMWCs) [51]. For chitosan depolymerization, nonspecific enzymes, such cellulase, hemicellulase, lysozyme, pectinase, and a-amylase, were also used. Due to the structural similarity of chitosan and cellulose, polymers of D-glucose linked by b-1,4-glycosidic linkages, many researchers have concentrated on cellulase to produce COS from chitosan. It appears that the enzyme does not strongly recognize the group at the C-2 position in glucose or glucosamine residues when the enzyme–substrate complex is formed [52].

### 3.3. Oxidative Degradation

By using free radicals formed by oxidants in aqueous solutions, oxidative degradation attacks the chitosan’s β -1,4 glycosidic bonds with active amino groups [53]. The response surface approach using 5.5% hydrogen peroxide chitosan can be converted into COS with a yield of 93.5% [54]. According to a first-order kinetics study, higher temperatures and concentrations result in prolonged reaction times and chitooligosaccharides with a higher mono-to-disaccharide-yield ratio [53]. The reduction in active amino groups and the browning of the product during the later stages of the degradation process are two drawbacks of the H_2_O_2_ degradation method. Two COSs with DDs of 69% and 95% can be separated from the same source material using a mixture of H_2_O_2_ and acetic acid in a ratio of 1:7 [55]

### 3.4. Electrochemical Degradation

The electrochemical degradation of COS is an innovative method for its preparation. This method is simple and contaminant-free but has significant drawbacks, such as a short electrode life and a high failure rate [56,57]. The protonated amino groups remained stable after electrochemical treatment, and the molecular weight of chitosan decreased with an increase in the current density of the Ti/TiO_2_-RuO_2_ electrode [56]. In addition, the Ti/Sb-SnO_2_ electrode was observed to be more effective than the Ti/TiO_2_-RuO_2_ electrode due to the difference between electrode materials’ characteristics [57].

## 4. Therapeutic Applications of Chitooligosaccharides

**Figure 4:**
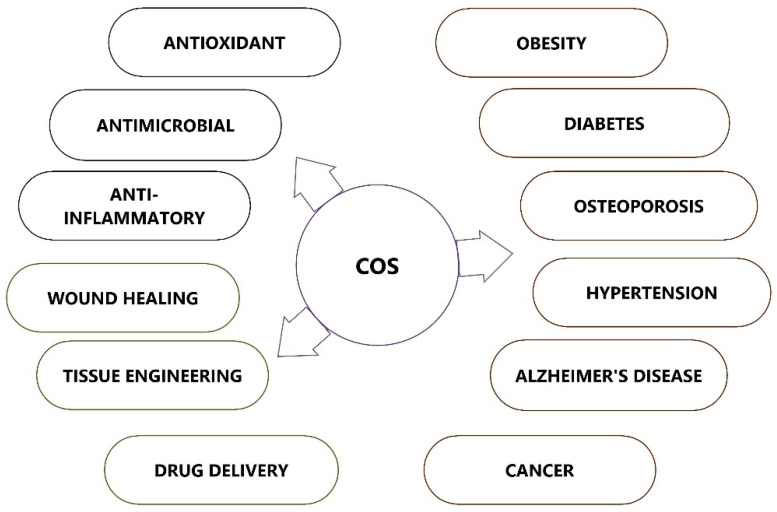
Therapeutic applications of chitooligosaccharides (COSs).

COS possesses many biological activities and promising applications in multiple fields, such as medicine, cosmetics, food, and agriculture (Figure 4). COS, which is recognized as low MW and water soluble, is in much greater demand than its precursor molecule chitosan. This section attempts to describe several biological activities of COS that can be applied in the field of medicine.

### 4.1. Chitooligosaccharides as Antioxidant Agents

Antioxidants can scavenge free radicals and protect the human body from highly toxic reactive oxygen species (ROS), slowing the progression of many chronic diseases. The ability of chitosan and its derivatives to scavenge free radicals and prevent oxidative damage by interrupting radial chain reactions is well established. Compared to chitosan, COS and its derivatives have better antioxidant properties [58,59]. COS antioxidant or radical-scavenging properties are primarily determined by their molecular weights and DD [46,58,60]. Several chronic diseases, such as cardiovascular diseases, atherogenesis, cancer, and Parkinson’s disease, are all linked to oxidative stress [61]. The excessive generation of ROS damages proteins, lipids, and DNA, leading to inflammation, tissue degeneration, and cellular apoptosis. ROS plays a vital role in the wound healing process at low concentrations, and an excessive level of reactive oxygen species can hinder wound healing by stimulating processes, such as inflammation and fibrosis [62]. 

Though the molecular mechanism underlying COS scavenging activity is unknown, the presence of hydroxyl and free amino groups in COS is thought to be responsible for its antioxidant activity. Antioxidants are noted for their positive benefits on health by protecting cells from the harmful effects of oxidation. It was demonstrated that COS and its derivatives have a high overall reducing power and can effectively remove hydroxyl radicals and superoxide anions [63]. The COS scavenging mechanism is believed to be based on the reaction of hydroxyl and superoxide anion radicals with active hydrogen atoms in COS, producing stable macromolecule radicals [59,64]. As COS can provide positrons to free radicals and transform them into more stable products, it can interrupt the chain reaction caused by free radicals [65].

In human lung epithelial A549 cells, the gallic acid-conjugated COS was tested for its antioxidant and anti-inflammatory properties. Gallate-COS was discovered to have remarkably high DPPH radical scavenging capacity and protect against DNA damage by H_2_O_2_. This study observed that adding gallic acid to COS enhanced its anti-inflammatory and antioxidant capabilities [66]. The findings indicated that gallate-COS might be a potential preventive agent for lung inflammation and lung cancer brought on by free radicals. In a high-fat-diet mouse model, the ability of COS to scavenge free radicals was examined both in vitro and in vivo. It was discovered that COS had strong antioxidant properties that could shield animals from oxidative stress [67]. Catalase, superoxide dismutase, and glutathione peroxidase were significantly increased in the stomachs, livers, and sera of mice when COS was used in conjunction with a high-fat diet. This indicates that COS may possess some antioxidant properties and be able to restore the function of the enzymes that a high-fat diet compromises.

### 4.2. Chitooligosaccharides as Antimicrobial Agents 

COS is an effective antibacterial agent that inhibits the growth of various microbes, such as bacteria, fungi, and viruses [68,69]. The antimicrobial activity of COS depends on the molecular weight (MW), degree of polymerization (DP), and pattern of acetylation (PA), as well as the type of organism. Though the actual mechanism of the antimicrobial activity is not clearly understood, low-molecular-weight chitosan can penetrate bacterial cell walls, bind with DNA, and inhibit DNA transcription and mRNA synthesis [70]. Conversely, chitosan with a high molecular weight can bind to the negatively charged elements of the bacterial cell wall. As a result, it modifies the permeability of the cell, creates an impermeable layer around it, and prevents transport into the cell [71,72]. 

The acetylated sequences in the COS structure are necessary for antibacterial action, and COS with a greater number of acetylated sequences and fewer free amino groups possesses improved antimicrobial activity [73]. It was observed that Gram-positive and Gram-negative bacteria have varied responses to the antibacterial action of COS. Gram-negative bacteria have a negatively charged cell surface; hence, the positively charged amine group in COS can severely impede their growth. However, COS does not prevent the growth of Gram-positive bacteria very effectively. The interaction between positively charged amino groups of COS and negatively charged carboxylic acid groups of bacterial cell surfaces paves the way for the formation of polyelectrolyte complexes, resulting in the formation of an impermeable coating around the bacterial cell and the suppression of metabolic activity [74]. As a result of COS antibacterial properties, a lower pH value and more polymerization have also been deemed advantageous [75]. Chitooligosaccharides significantly block *A. actinomycetemcomitan* growth due to its effect on cell membrane permeability. The release of cellular components and the unregulated entry of substances from the surrounding environment results in microbial cell death [69]. 

COS antimicrobial activities are influenced by a variety of factors, including deoxycholic acid (DA) or dicetyl phosphate (DP), as well as other physicochemical properties and microorganism types [76]. COS can change the permeability characteristics of microbial cell membranes, preventing materials from entering or triggering the cell component’s leakage, ultimately leading to bacterial destruction. The bacterial envelope serves as the active site for COS, and membrane rupture may lead to the destruction of the microorganism. Chitosan penetration into bacterial DNA suppresses RNA transcription, an additional mechanism for killing microorganisms [77]. 

COS has antibacterial properties that promote tissue granulation and collagenase activity, two key factors in wound healing. Therefore, the antibacterial property of COS is responsible for its potential to promote wound healing. The antibacterial effects of COS are superior against both Gram-positive and Gram-negative microorganisms [78]. Antimicrobial action is enhanced with higher degrees of deacetylation than lower acetylation levels [79]. In a study conducted in relation to yeast, chitooligosaccharides with a degree of polymerization of 32 showed strong inhibitory efficacy [80].

*Streptococcus mutans*, a Gram-positive bacterium often observed in human dental caries, was susceptible to the antimicrobial action of chitooligosaccharide coupled with glycidyl trimethyl ammonium chloride after only two hours of exposure [81]. The antimicrobial activity of COS can also be enhanced by incorporating other nanomaterials. The impact of enhanced antibacterial potential was observed when the quaternary ammonium compounds of COS were added to zinc oxide and a palygorskite nanocomposite [82]. COS-coated silver nanoparticles showed efficient antimicrobial activity [83]. COS-coated silver nanoparticles with calcium hydroxide were tested against *Streptococcus mutans, Staphylococcus aureus,* and *Enterococcus faecalis* by Rajasekaran et al. [84]. The composite paste inhibited the investigated bacteria more efficiently, which could benefit pediatric pulp therapy.

An analysis of the antifungal activity of chitooligosaccharide with an average polymerization degree of 32 was observed to be effective against human fungal infections [80]. A study by Jang et al. [85] revealed that the low-molecular-weight chitooligosaccharide decreased the fluorescence intensity of the SARS-CoV-2 nucleocapsid protein of the virus-infected cells in a dose-dependent manner. They concluded that chitooligosaccharide can have an antiviral effect on SARS-CoV-2 and may be a potent natural treatment for COVID-19.

### 4.3. Chitooligosaccharides as Anti-Inflammatory Agents

Inflammation plays a crucial role in the pathology of various diseases, such as chronic asthma, rheumatoid arthritis, multiple sclerosis, inflammatory bowel disease, psoriasis, and cancer [86,87,88,89]. It has been demonstrated that the oral administration of COS inhibits the activation of myeloperoxidase, cyclooxygenase (COX)-2, and inducible nitric oxide synthase (iNOS), as well as the levels of proinflammatory cytokines, such as interleukin (IL)-6 and tumor necrosis factor (TNF)-α [90]. COS exposure may inhibit the generation of numerous proinflammatory cytokines associated with lipopolysaccharide (LPS)-induced inflammation without impairing cell viability [91]. NF-κB (nuclear factor kappa-light-chain-enhancer of activated B cells) nucleus translocation was decreased as a result of the COS suppression of LPS-induced inflammatory gene expression, which attenuated an LPS-induced inflammatory response in vascular endothelial cells [92]. Ma et al. [93] hypothesized that COS could reduce the phosphorylation levels of mitogen-activated protein kinases (MAPKs) and activate NF-κB and activator protein 1 (AP-1) to decrease the LPS-induced interleukin 6 (IL-6) and TNF-α generation in macrophages. In a study on the effects of COS on synoviocytes in rabbit knee joints, it was found that activating AMPK reduced the expression of the enzymes iNOS and COX-2, thereby reducing synovial inflammation [94].

COS supplementation in the diets of animals has been shown to have a potent immunoenhancing effect. The results obtained from an evaluation of the COS effect on cyclophosphamide-induced immunosuppression by Mei et al. [95] showed that in mice treated orally with COS and cyclophosphamide, the delayed-type hypersensitivity reaction, macrophage phagocytosis activities, and levels of cytokines IL-2, IL-12, and interferon were significantly increased, while the production of IL-10 was decreased. COS exhibited protective benefits against ovalbumin-induced lung inflammation in induced asthmatic mouse models at a maximal dose of 16 mg/kg per day, with a significant decrease in mRNA expression and protein levels of IL-4, IL-5, IL-13, and TNF-α in lung tissue and bronchoalveolar lavage fluid [96]. The serum levels of IL-1, IL-2, IL-6, immunoglobulin (Ig) A, IgG, and IgM were all raised in early weaned pigs when COS was reintroduced into their diets. Increased cell-mediated immunity in response to weaning stress may be possible if COS can modulate the levels of specific cytokines and antibodies [97]. COS inhibits the release of nitric oxide in LPS-induced RAW 264.7 cells and BV-2 microglia, diminishes the glycerol-induced inflammatory response in rat kidneys, and decreases organ failure in LPS-induced sepsis [98,99].

Glycoprotein YKL-40 promotes angiogenesis and functions as a negative regulator of the inflammasome. The high-affinity binding of N-acetylglucosamine to YKL-40 and subsequent stimulation of chondrocyte proliferation could be used as a novel therapeutic strategy for treating inflammatory rheumatoid diseases [100]. In treating rheumatoid arthritis, it may be beneficial to elucidate the proinflammatory mechanism of COS [101]. COS could be considered as a new functional diet for IBD patients based on the observations from the animal study. Orally administered COS decreased the shortening of the colon length and tissue injury in mice and controlled inflammation in the colonic mucosa [90].

These research findings based on the above-mentioned hypothesis indicate that COS can induce an anti-inflammatory effect by inhibiting cyclooxygenase and reducing prostaglandins. However, additional research is required to determine the effect on more extended inflammatory periods and the bioavailability of these substances.

### 4.4. Chitooligosaccharides and Their Anti-Obesity Activity

Obesity is a chronic trophic metabolic disorder primarily caused by an energy imbalance, resulting in the buildup of excess body fat. Obesity is associated with type 2 diabetes (T2D), hyperlipidemia, hypertension, cerebrovascular events, and cancer [102]. COS has excellent water solubility and lower viscosity than chitosan, and the intestine more easily absorbs it. In animal models, COS has also been shown to induce weight loss, lower triglyceride and cholesterol levels in serum, and prevent lipid buildup in hepatocytes and adipose tissues [103,104]. COS has demonstrated enhanced intestinal absorption, and studies have been performed to investigate its potential to reduce weight gain, blood triglyceride/cholesterol levels, and lipid buildup in the liver and adipose tissues [25,105,106]. COS anti-obesity activity has been the subject of numerous hypotheses, but its precise mechanism has not been fully elucidated. For instance, COS may block enzymes or interact with bile acids, resulting in decreased fat absorption and increased fecal fat excretion [107].

When obese rats were fed COS, higher amounts of high-density lipoproteins or cardioprotective lipid-containing particles were discovered than in control rats. These lipoproteins and particles are responsible for removing excess cholesterol from tissues and transporting them to the liver [103]. Low-MW COS appears to be more effective in increasing plasma and hepatic lipoprotein lipase activity [108]. The evidence obtained using mouse models suggested that COS inhibited the expression of apolipoprotein B, which reduced the amount of cholesterol found in the serum [109]. Comparing the anti-obesity activity of COS and resistant starch (RS) to their combination, COS–RS, in rat models of induced obesity and dyslipidemia, COS–RS displayed the most significant fat and lipid-lowering benefits, followed by COS and RS [110]. Their examination of RNA sequencing revealed an increased conversion of cholesterol to bile acids. COS reduces triglycerides, total cholesterol, low-density lipoprotein cholesterol serum levels, and the expression of endoplasmic reticulum stress pathway-related factors (GRP78, GRP94, ATF4, and CHOP), and increases oxidative lipid catabolism [111]. This effect may be mediated by metabolites or directly by the molecules themselves. These findings underline the significance of developing COS as a potential drug derived from natural products for the prevention and treatment of obesity.

### 4.5. Chitooligosaccharides as Antidiabetic Agents

COS has demonstrated the potential to protect β cells from excessive glucose by promoting pancreatic cell proliferation, resulting in enhanced insulin production to lower glucose levels [112,113]. Reducing blood glucose levels and restoring normal insulin sensitivity were among the documented benefits of COS therapy for diabetic rats [113]. COS possesses antidiabetic properties and may reduce diabetes mellitus (DM) incidence by affecting the glucose–lipid metabolic balance and glycemic control [114]. Additionally, COS therapy can potentially improve the general health of diabetic rats, alleviate diabetic symptoms, bring blood glucose levels back to normal, and restore normal insulin sensitivity.

In addition, chitooligosaccharides can stimulate the multiplication of beta cells and restore the functional capacity of injured beta cells. COS has been shown to promote the overgrowth of beta cells and isolated pancreatic islet cells, in addition to increasing insulin release from pancreatic cells [113]. Chitooligosaccharides have the potential to significantly increase the rate of proliferation of pancreatic islet cells. In diabetic mice, it was also found that dietary COS reduced hyperglycemia by activating hepatic glucokinase and increasing peripheral tissue glucose uptake, as well as by increasing pancreatic insulin secretion and improving skeletal muscle glucose uptake [115]. In a study using streptozotocin (STZ)-induced diabetic rats, COS was able to treat hyperglycemia at a dose of 1000 mg/kg by lowering fasting serum glucose and insulin levels, thus improving O-glycosyltransferase (OGT), enhancing the index of insulin sensitivity, and reducing insulin resistance [116]. Additionally, COS significantly increases the amount of glycogen in the liver by increasing glucokinase, which facilitates the transfer of blood glucose into liver glycogen. Low-molecular-weight COS also increases the plasma adiponectin levels in prediabetic subjects [117]. 

Significantly elevated serum glutamic pyruvic transaminase (SGOT) and serum glutamic pyruvic transaminase (SGPT) activities were observed in alloxan-induced diabetic mice. As blood levels of amino acids increase, gluconeogenesis and ketogenesis increase, which results in enhanced transaminase activity in the absence of insulin [118]. After receiving COS (10 mg/kg), the liver function indicators SGOT and SGPT returned to normal levels, indicating that the liver was functioning normally.

Chronic hyperglycemia may be acquired, consequent to the failure of peripheral tissues to utilize glucose properly. Glucose transporter type 4 (GLUT-4) is a vital glucose transporter and regulator of its metabolism found in skeletal muscles and adipocytes. The expression of GLUT-4 was decreased in adipose and skeletal tissues in a diabetic animal model [119]. The treatment of the diabetic animals with COS caused a significant increase in GLUT-4, which might upregulate GLUT-4 mRNA expression, thereby improving insulin resistance. COS may have therapeutic effects on type 2 diabetes due to its capacity to boost liver glycogen production and GLUT-4 gene expression in soleus muscle and adipose to ameliorate insulin resistance. COS, on the other hand, exhibited an antidiabetic impact by stimulating proliferation, increasing insulin release and GLUT-2 mRNA levels, and protecting against STZ-induced apoptosis in β cells. In addition, COS activities, such as enzyme inhibition, antioxidant activity, and immunomodulation, may assist non-obese diabetic mice in avoiding the development of type 2 diabetes mellitus, which promotes the preservation of pancreatic β cells and the standardization of the vital insulin secretion [120].

### 4.6. Chitooligosaccharides in Osteoporosis

Supplemental chitooligosaccharides are beneficial in calcium-deficient states, such as osteoporosis. Supplementing animals’ diets with COS has been shown to improve calcium bioavailability in rat osteoporosis models induced by ovariectomy and concomitant low calcium intake [121]. A substantial reduction in the serum levels of inflammatory cytokines was observed after the oral administration of COS to older persons [122]. The mechanism behind the possible antiosteoporotic effect of COS was thought to be connected to its anti-inflammatory properties. Additionally, others have reported that COS suppressed the synthesis and expression of proinflammatory mediators in vitro [121,123].

The antiosteoporotic effect of COS may be partially explained by its anti-inflammatory effect via the downregulation of COX-2 expression levels, which provides additional evidence for the efficacy of selective COX-2 inhibition in preventing bone loss in estrogen-deficient animals and postmenopausal women [124]. The mineralization process and bone density depend on Ca^2+^, which provides structural support. There is evidence that COS causes an increase in bone calcium deposition [121,125]. Jung et al. [121] discovered that COS effectively inhibited the development of insoluble calcium-phosphate salts, enhancing Ca^2+^ bioavailability and bone strength. COS (5 kDa) enhanced calcium retention and lowered bone turnover in a rat osteoporosis model, indicating that COS may have favorable effects as a calcium supplement in Ca^2+^ shortage, such as osteoporosis.

### 4.7. The Antihypertensive Effect of COS

COS can effectively control hypertension by inhibiting renin or angiotensin-converting enzyme activity. COS is an angiotensin-converting enzyme (ACE) inhibitor since the active binding site of ACE is positively charged and contains hydrogen-bond acceptors and zinc as a cofactor. Hong et al. [126] evaluated the ACE inhibitory actions of COS with varying degrees of polymerization from 1 to 10 and found that the chitotriose (DP = 3) derivative was the most potent. Studies have shown that the DD is inversely proportional to the ACE inhibitory activity of COS or its derivatives [127,128]. Carboxylated and sulfated COS have been synthesized, and it has been observed that they possess a significantly greater inhibitory action against ACE than unmodified COS [127,128]. It is believed that an increase in negative charges on the molecule is responsible for the improved binding of this modified COS to the integral active site of the enzyme, thereby boosting its ACE inhibitory action. The aminoethyl-conjugated COS exhibited enhanced ACE inhibitory action due to the formation of hydrogen bonds, which promotes COS binding [129]. 

### 4.8. Chitooligosaccharides and Alzheimer’s Disease

The pathological changes in Alzheimer’s disease (AD) are caused by neuronal apoptosis, and neuronal protection is crucial in treating it [130]. A study conducted to explore the neuroprotective effect of COS against AD showed that COS significantly decreased amyloid beta-induced cell apoptosis by reducing the expression of caspase 3 and Bax/Bcl-2 ratio activation [131]. COS significantly reduced the neuronal damage caused by oxidative stress and glucose deprivation, and these findings imply that COS has the potential to serve as a neuroprotective agent against neurodegenerative diseases, such as Alzheimer’s disease [132]. The protein expression and acetylcholinesterase activity produced by amyloid β peptide in PC12 cells were inhibited by COS, which was revealed to be the first stage in the pathogenic cascade of AD [133]. A mouse model demonstrated that COS could be a valuable drug for treating sciatic nerve injury and reducing scar tissue formation [134]. Evidence shows that COS could have neuroprotective effects by reducing oxidative stress and neuroinflammatory responses [135].

### 4.9. Chitooligosaccharides as Antitumor Agents

**Figure 5:**
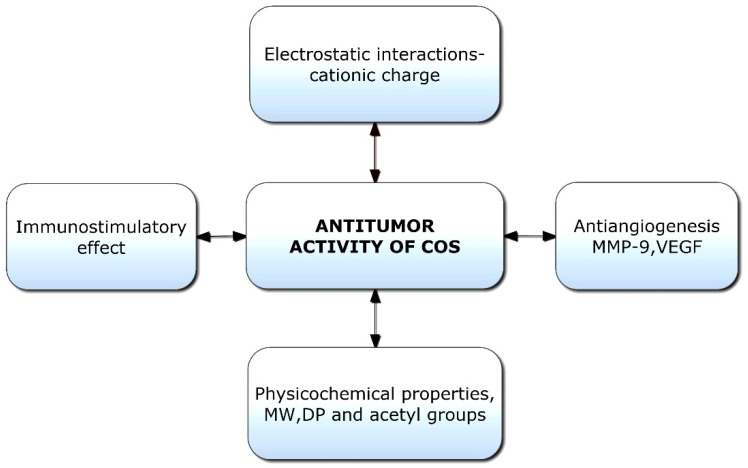
Potential factors responsible for antitumor activities of chitooligosaccharides (COSs). VEGF: Vascular endothelial growth factor; MW: Molecular weight; DP: Degree of polymerization.

Chitooligosaccharides and their derivatives have demonstrated potent antitumor activity against human cancer cells [136]. Though limited information is available on the antitumor mechanisms of COS, some hypotheses have been proposed (Figure 5). Initially, the mechanism of anticancer activity was linked to the cationic character of COS; however, it was later hypothesized that relative molecular weight was also a crucial factor [137]. The tumor-inhibiting effect of COS is likely a result of its ability to induce lymphocyte cytokines via promoting T-cell proliferation. The antitumor mechanism of COS is essentially enhanced by acquired immunity by increasing T-cell differentiation to boost cytotoxicity and preserve T-cell activity [138]. COSs are naturally occurring polysaccharides with a cationic charge; they can promote the apoptosis of numerous cancer cells, including liver cancer, breast cancer, cervical cancer, kidney cancer, lung cancer, leukemia, and colorectal cancer cells [25]. COS with a cationic charge can absorb tumor cells by modifying their permeability through electrostatic contact. The binding of COS to tumor cell membranes and subsequent changes in the ionic environment cause alterations in the permeability of tumor cells [139]. However, due to the mutual repulsion between COS and healthy cells with a uniform positive charge, only tumor cells are targeted, not healthy ones.

The antitumor activity of COS is linked to its ability to stimulate cytokine production through enhanced T-cell proliferation. It was established that COS could prevent tumor growth via immune-boosting actions [140]. Immunostimulatory activities of chitooligosaccharides indirectly contribute to tumor suppression by boosting the generation of lymphokines, inducing lymphocyte factors, and stimulating the proliferation of cytolytic T lymphocytes, hence producing an antitumor effect [95,141]. Furthermore, COS can inhibit tumor angiogenesis by inhibiting the expression of matrix metalloproteinase-9 (MMP-9), which in turn inhibits the expression of vascular endothelial growth factor (VEGF) [142]. MMP-9 is an essential component in cancer invasion and metastasis, and the upregulation of MMP-9 expression may lead to the release of VEGF and the formation of angiogenesis [143]. Additionally, by inhibiting the production of VEGF and urokinase-type plasminogen activator in vascular endothelial cells, COS increases its ability to inhibit tumor angiogenesis [144].

Aminoethyl-chitooligosaccharides are made by replacing the hydroxyl group at C-6 with an aminoethyl group, and they were found to inhibit cell invasion and metastasis [145]. Necrosis was also found to be the principal mechanism by which a highly charged COS derivative inhibits the growth of cancer cells, according to a study analyzing DNA fragments [139]. However, low-molecular-weight COS and *N*-acetyl-d-glucosamine oligomers (NACOS) can potentially cause apoptosis in cancer cells [146,147]. COS also inhibits tumor progression by modulating key pathways in malignant growth, such as the suppression of β-catenin, mTOR, pyruvate kinase, and ornithine decarboxylase; the activation of caspase 3 in tumor cells; and the stimulation of interferon gamma (IFN-γ) and IL-12 secreted by natural killer (NK) cells [148].

Several potential mechanisms were explained based on the animal studies. COS-based drug delivery and therapy systems for anticancer applications have great potential if novel synthesis methodologies and a cost-effective strategy can be developed. Future research is required to investigate the molecular mechanism of COS on cancer cells and its effect on signaling pathways.

### 4.10. Chitooligosaccharides in Wound Healing 

Hemostasis, inflammation, proliferation, and remodeling are some stages of the wound healing process that are influenced by the immunostimulating properties of materials that aid in healing, such as proinflammatory cytokines, inflammatory cells, and growth factors [149]. As a result, the demand for biomaterials with superior biological properties, such as antibacterial, antioxidant, and anti-inflammatory properties, has increased exponentially. The improved biological properties of COS, including cytocompatibility, antibacterial, antioxidant, anti-inflammatory, and immunostimulatory activities, may speed up the wound healing process. As a result of its beneficial biological actions, including antimicrobial protection, increased permeability to air and moisture, cellular adhesion, and cell proliferation, COS is a promising therapeutic agent in the treatment of tissue damage and wound healing.

The overexpression of miR-27a and the activation of the transforming growth factor-beta (TGF-β)-1-Smad2/3 pathway are two potential mechanisms of COS in the acceleration of wound healing and tissue regeneration [150]. COS is combined with other biopolymers for wound healing due to its low molecular weight. In addition to its biological properties, COS can promote wound healing by enhancing a wound dressing’s water absorption, flexibility, and mechanical strength.

However, the ability of COS to heal wounds is affected by its molecular weight and degree of acetylation [151]. The local injection of immunostimulatory COS into wounds could stimulate the production of growth factors and the migration of keratinocytes, both of which are essential for wound healing [152]. COS, which shares the same chain length as oligonucleotides, possesses higher immunostimulatory activity and enhances the activity of macrophages. In COS-dressed wounds, the more significant migration of macrophages to injured tissues stimulates the development of type-III collagen, expediting the healing process.

The wound healing activity of oxidized alginate-gelatin hydrogel was enhanced using COS and salicylic acid conjugates. Collagen proliferation at the wound sites was accelerated by the COS conjugate, leading to quicker healing [153]. Applying COS to the locations of sciatic nerve lesions demonstrated the drug’s multifaceted impact on healing. The restoration of nerve function was accelerated by COS because it decreased the ratio of type I to III collagen, which boosted axon regeneration and slowed axonal demyelination. COS was discovered to slow the proliferation of fibroblasts, an essential component of scar tissues that prevents axonal regeneration [134,151]. COS has been found to induce the formation of type I and III collagen in wounds, repair injured nerves, expedite hemostasis, and protect against infections, making it an effective wound healing agent [153,154]. Combining medications, such as curcumin with COS, enhances their therapeutic efficacy [155]. COS is, therefore, superior to chitosan because it possesses a more potent biological activity without modification [153,154].

### 4.11. Chitooligosaccharides in Tissue Engineering

Cells, composite scaffolds, and signaling molecules are the three essential components for producing substituted tissues that repair, replace, or regenerate damaged tissues or organs. COS is comparable to glycosaminoglycans, a crucial part of the extracellular matrix of a cell (ECM). As it can create an environment that closely resembles the ECM, allowing for cell attachment and the preservation of growth factors, this makes it successful for scaffolds in tissue engineering applications [156]. In the creation of bone tissue engineering scaffolds, natural polymers, such as gelatin and COS, have been used. By regulating the genes that control osteoblast proliferation in bone tissues, COS has been shown to stimulate neuronal differentiation in PC-12 nerve cells [157]. Gelatin–COS scaffolds that were cultivated with mesenchymal stem cells (MSCs) obtained from bone marrow and capable of osteogenic differentiation demonstrated promising outcomes in the production of bone tissue [158]. Within two weeks of implantation, cell proliferation was observed inside the scaffolds; homogenous collagen distribution within the pores and calcium deposition on the scaffolds’ surfaces indicated that the cells had successfully proliferated [159]. An increase in COS content caused the cross-linking density to decrease, which allowed the scaffolds to swell to more than twice their dry size and made them more permeable to cells. The COO−, C—O, and amino groups present in COS serve as nucleating sites for hydroxyapatite crystals [158].

Single-walled carbon nanotubes (SWCNTs) have been added to synthetic composite materials as alternatives to bone grafts. By enhancing SWCNT implants with organic polymers, such as chitosan and COS, it is possible to keep the graft in constant contact with the tissues, which speeds up bone tissue formation and eliminates the need for additional procedures [160]. A collagen the COS scaffold that was created to act as a synthetic skin tissue was discovered to have improved resistance to collagenase and other deteriorating enzymes. Collagenase would quickly degrade artificial collagen if used alone; hence, collagen was combined with COS to create densely linked microporous structures with enhanced mechanical characteristics [161]. In contrast to pure collagen scaffolds, scaffolds made with COS showed a higher rate of fibroblast growth.

### 4.12. Chitooligosaccharides in Drug Delivery

COS has excellent potential for usage in drug delivery systems (DDSs) due to its non-toxicity, biodegradability, and solubility in water [154]. Due to its solubility at physiological pH, the water solubility of COS is generally more effective for drug administration [162]. Recently, a novel alternative to red blood cells has been developed using a pectin-based COS–hydrogel microcapsule carrier designed to transport hemoglobin [163]. Oligosaccharides were used to extend the shelf life of these therapeutic drugs by several months. With COS of a 95% degree of deacetylation (DD) and an MW of 10 kDa, non-biodegradable polyethylene glycol (PEG) and cyclodextrin inclusion complexes are converted into hydrogels capable of transporting drugs.

MW and DD are influential parameters for modulating the actions of COS, offering a strategy to improve its efficacy in drug delivery systems (DDSs) [164]. COS is more significantly absorbed through negatively charged mucous membranes of tissues because it is more capable of binding under these situations [165]. DD also controls binding; at a low MW, the effect of DD on charge density is more substantial [28,166]. This may have a positive influence on the interaction of COS with oppositely charged copolymers or active medicines; nevertheless, the considerably stronger interactions can harm cell viability [167]. Therefore, when utilizing COS with a high DD in drug delivery, it is necessary to monitor and optimize the dosage rate to limit cytotoxic effects [164].

After combining it with the aqueous copolymer solution, COS is used as a hydrogel precursor to speed up the production of cyclodextrin hydrogel, which can be completed in as little as five minutes. Due to the synergistic interaction between hydrated COS and hydrophobic aggregated inclusion complexes of cyclodextrin with an MPEG-PCL graft and unbound PCL blocks, rapid gelation resulted in the formation of a stable supramolecular hydrogel. This was attributed to the hydrogel containing unbound PCL blocks [168]. Mahato et al. (2020) created hydrogel systems based on PVA and COS for drug delivery applications. In addition, the antibacterial agent lomefloxacin was added to the hydrogels, and sustained drug release was observed [169].

The decrease in chitosan’s molecular weight increases its affinity for polyanionic complexes and mucosal membranes. This may increase the uptake rate of COS-based DDS through targeted tissues due to the substance’s increased retention at the location [170]. Garaivo et al. [171] determined that chitosan oligomers are superior to chitosan for the intracellular delivery of genes because they are not taken up by endocytic vesicles coated with the clathrin protein, which transfers the polyplexes to the lysosome for degradation. This is the fate that linear chain chitosan-DNA nanoplexes typically face. The graft copolymer is created by graft–diblock copolymers of amphiphilic polyethylene glycol methyl ether and polycaprolactone onto hydrophilic COS.

## 5. Conclusions and Future Prospects

Chitooligosaccharides are derived from the deacetylation and depolymerization of chitin or chitosan through physical, chemical, or enzymatic hydrolysis. Though several methods can be used to convert chitin and chitosan, the lack of a standard method to obtain highly pure COS with a stable DP for large-scale applications is challenging. COS with a varying MW, DP, and concentrations has been shown to possess various therapeutic effects, including antimicrobial, antitumor, immunoregulatory, and antioxidant activities, which can potentially treat and prevent diseases, such as cancer, diabetes mellitus, cardiovascular disease, and infectious diseases. In addition, due to the cationic sphere on the more exposed shorter N-glucosamine units, COS is absorbable through the intestinal epithelium and displays a greater amount of cellular transduction. Despite its widespread use, its stability, safety, and purity have not been proved successful. At present, limited information is available on the definitive impacts of the physicochemical properties of COS on various biological functions and the molecular mechanisms underlying bioactivities. Examining the molecular structure of COS, which is accountable for its biological activity, is essential for comprehending its process. Several studies on the biological activities of COS and its toxicities were performed in cell and animal models and using COS mixtures or purified COS with insufficiently defined chemical characteristics. Future investigations and translational research should investigate COS therapeutic effects and toxicity profiles with well-defined chemical characteristics in either large animal models or human subjects. 

## Data Availability

Not applicable.

## Abbreviations

ACE	Angiotensin-converting enzyme
LPSs	Lipopolysaccharides
AD	Alzheimer’s disease
MAPKs	Mitogen-activated protein kinases
AP-1	Activator protein 1
MMP-9	Matrix metalloproteinase 9
COS	Chitooligosaccharide
MSC	Mesenchymal stem cell
COX	Cyclooxygenase
MW	Molecular weight
DA	Deoxycholic acid
NaBH4	Sodium borohydride
DD	Deacetylation degree
NACOSs	N-acetyl-d-glucosamine oligomers
DD	Degree of deacetylation
NaNO_2_	Sodium nitrite
DDSs	Drug delivery systems
NF-κB	Nuclear factor kappa-light-chain-enhancer of activated B cells
DM	Diabetes mellitus
NK	Natural killer
DP	Degree of polymerization
OGT	O-glycosyltransferase
ECM	Extracellular matrix
PEG	Polyethylene glycol
GlcN	Glucosamine
ROS	Reactive oxygen species
GlcNAc	N-Acetylglucosamine
RS	Resistant starch
GLUT-4	Glucose transporter type 4
SGOT	Serum glutamic pyruvic transaminase
H_2_O_2,_	Hydrogen peroxide
SGPT	Serum glutamic pyruvic transaminase
HCl	Hydrochloric acid
STZ	Streptozotocin
IFN-γ	Interferon gamma
SWCNTs	Single-walled carbon nanotubes
IL	Interleukin
TGF-β	Transforming growth factor beta
iNOS	Inducible nitric oxide synthase
TNF	Tumor necrosis factor
LMWCs	Low-molar-mass chitosans
VEGF	Vascular endothelial growth factor
